# Dysregulation of the CD163-Haptoglobin Axis in the Airways of COPD Patients

**DOI:** 10.3390/cells11010002

**Published:** 2021-12-21

**Authors:** Andrew Higham, James M. Baker, Natalie Jackson, Rajesh Shah, Simon Lea, Dave Singh

**Affiliations:** 1 Division of Immunology, Immunity to Infection and Respiratory Medicine, School of Biological Sciences, Faculty of Biology, Medicine and Health, Manchester University NHS Foundation Trust, Manchester M23 9LT, UK; james.baker-6@postgrad.manchester.ac.uk (J.M.B.); simon.lea@manchester.ac.uk (S.L.); dsingh@meu.org.uk (D.S.); 2 Medicines Evaluation Unit, Manchester M23 9QZ, UK; njackson@meu.org.uk; 3 Department of Thoracic Surgery, Manchester University NHS Foundation Trust, Manchester M23 9LT, UK; rajesh.shah@mft.nhs.uk

**Keywords:** iron, airway inflammation, bacteria, haemoglobin, oxidative stress, macrophages, eosinophils, smoking, inhaled corticosteroids, sputum

## Abstract

Pulmonary iron levels are increased in chronic obstructive pulmonary disease (COPD) patients. Iron causes oxidative stress and is a nutrient for pathogenic bacteria. Iron may therefore play an important role in the pathophysiology of COPD. The CD163-haptglobin axis plays a central role in the regulation of iron bioavailability. The aim of this study was to examine dysregulation of the CD163-haptglobin axis in COPD. We measured soluble CD163 (sCD163) and haptoglobin levels in sputum supernatants by enzyme-linked immunosorbent assay (ELISA) and sputum macrophage CD163 and haptoglobin expression by flow cytometry in COPD patients and controls. SCD163 levels were lower in COPD patients compared to controls (*p* = 0.02), with a significant correlation to forced expiratory volume in 1 s (FEV1)% predicted (rho = 0.5, *p* = 0.0007). Sputum macrophage CD163 expression was similar between COPD patients and controls. SCD163 levels and macrophage CD163 expression were lower in COPD current smokers compared to COPD ex-smokers. Haptoglobin levels were not altered in COPD patients but were regulated by genotype. Macrophage CD163 and haptolgobin expression were significantly correlated, supporting the role of CD163 in the cellular uptake of haptoglobin. Our data implicates a dysfunctional CD163-haptoglobin axis in COPD, which may contribute to disease pathophysiology, presumably due to reduced clearance of extracellular iron.

## 1. Introduction

Chronic obstructive pulmonary disease (COPD) is a heterogeneous disease caused by the inhalation of noxious particles, commonly from cigarette smoking [1]. The typical pathological features include small airway inflammation, mucus hypersecretion, tissue remodelling and parenchymal destruction (emphysema) [2]. There are increased numbers of inflammatory cells in the lungs, including neutrophils, macrophages and lymphocytes. COPD macrophages display altered phenotypic features coupled with reduced efferocytosis and phagocytosis functions, with the latter contributing to bacterial colonisation [3,4].

Iron levels are increased in the lungs of COPD patients [5]. Cigarette smoke and red blood cells are potential sources of excess iron in the lungs [6]. Free iron is a catalyst for reactive oxygen species production and is an essential nutrient for pathogenic bacteria [7,8]. Iron may therefore play an important role in the pathophysiology of COPD.

Haptoglobin is an acute phase protein, with three genotypes: 1-1, 2-1 and 2-2. Individuals with 1-1 genotype have higher levels of serum haptoglobin compared to 2-1 and 2-2 individuals, suggesting haptoglobin levels are under genotype control [9,10]. Haptoglobin binds free haemoglobin to form the haptoglobin-haemoglobin complex [11,12]. The complex is endocytosed by macrophages via the CD163 receptor, whereby haemoglobin is metabolised to produce non-toxic iron [13]. Haptoglobin levels are increased in the serum of COPD patients, and a negative association with lung function has been reported [14], suggesting that dysregulation of haptoglobin expression is involved in COPD pathophysiology. Haptoglobin protein expression has been confirmed in lung macrophages and alveolar epithelial cells in COPD patients [11], while haptoglobin mRNA expression has been observed in lung eosinophils [15]. However, it is not known whether pulmonary haptoglobin levels are different in COPD patients compared to controls.

Several studies have examined CD163 expression in COPD lung macrophages. Eapen et al. found reduced numbers of CD163+ macrophages in the small airways of COPD patients with global initiative for chronic obstructive lung disease (GOLD) stages 1 and 2 severity compared to non-smoking controls, whereas there were increased numbers of CD163+ macrophages in the bronchoalveolar lavage of COPD patients compared to controls [16]. Kaku et al. demonstrated increased numbers of CD163+ macrophages in the alveolar space of COPD patients with GOLD stages 3 and 4 severity compared to non-smokers, smokers and COPD patients with stages 1 and 2 severity [17]. Overall, these two studies suggest upregulation of CD163 in COPD patients compared to controls, although the findings are not entirely consistent. There is also evidence that CD163 levels are reduced in interstitial and BAL macrophages from COPD current versus ex-smokers [18,19] and macrophage CD163 expression is regulated by corticosteroids [20].

CD163 is also present as a soluble form (sCD163), produced by cleavage of membrane bound CD163 by ADAM17 [21] or by release in extracellular vesicles [22]. SCD163 binds to haptoglobin–haemoglobin complexes to facilitate cellular uptake [23]. There is no published data describing sCD163 levels in the lungs of COPD patients.

The CD163-haptoglobin axis plays a central role in the regulation of iron bioavailability. Here, we measured levels of sCD163, CD163 and haptoglobin proteins in lung samples from COPD patients and controls to investigate potential dysregulation of the CD163-haptoglobin axis in COPD. We also investigated the effects of current smoking on the expression of these proteins.

## 2. Materials and Methods

### 2.1. Study Subjects

#### 2.1.1. Sputum Study

Ten healthy non-smokers (HNS), ten healthy smokers (HS) and seventeen COPD patients were recruited for sputum induction. HS were a mixture of current and ex-smokers with a >10 pack-year smoking history and normal spirometry. Ex-smokers were defined as individuals who had stopped smoking for ≥1 year. COPD was diagnosed based on GOLD recommendations [1]. COPD patients were a mixture of current smokers (COPDS) and ex-smokers (COPDE). All subjects had no history of respiratory illness or antibiotic use within six weeks of the study. This study was approved by NRES Committee North West—Greater Manchester East (ref code 05/Q1402/41).

#### 2.1.2. Resected Lung Tissue Study

Twenty-one patients undergoing surgical resection for suspected lung cancer were recruited for gene expression analysis. These were a mixture of COPDS and COPDE. This study was approved by NRES Committee North West–Greater Manchester South (reference 20/NW/0302).

All experiments were performed in accordance with relevant guidelines and regulations. This research has been carried out in accordance with the World Medical Association Declaration of Helsinki of 1975, and all subjects provided written informed consent.

### 2.2. Sputum Processing

Sputum was induced as previously described [24] using 3%, 4% and 5% saline, inhaled in sequence for 5 min, for a maximum of 15 min via an ultrasonic nebuliser (EASYneb II, Flaemnouva, Italy). To minimise contamination of saliva, all subjects were instructed to thoroughly rinse their mouth with distilled water and perform coughing prior to sputum expectoration. Sputum plugs were isolated from the saliva component, combined proportionately with phosphate buffered saline (PBS) and vortexed for 10 s, rocked for 15 min and centrifuged (790× *g* for 10 min at 4 °C). PBS supernatants were removed; 0.2% Dithiothreitol (DTT) was added, and the suspension was vortexed for 10 s, rocked for 15 min and filtered using a 48 µm filter (Sefar Ltd., Bury, UK). The suspension was centrifuged (790× *g* for 10 min at 4 °C), DTT supernatants were removed, the cell pellet was re-suspended in PBS and cytospins were prepared. Slides were air-dried for 30 min and then fixed in methanol for 10 min before staining with RapiDiff (Triangle, Skelmersdale, UK) for differential cell counting.

### 2.3. Flow Cytometry

Flow cytometry was carried out with 0.5 × 10^6^ sputum cells per tube. Cells were washed with wash buffer. Ice cold 2% Parafix (BD Biosciences, Oxford, UK) was then added and incubated at 4 °C for 10 min. Cells were then washed again. Perm Buffer I (BD Biosciences, Oxford, UK) was then added and incubated for 20 min at 4 °C, followed by a wash in Perm Buffer I. The following antibodies: haptoglobin (antibodies-online, ABIN952678 (primary), APC Conjugation Kit (secondary), Abcam, ab201807), CD163 (Thermo Fisher Scientific, Loughborough, UK 12-1639-42), CD206 (Biolegend, London, UK 321120) and CD45 (Biolegend, London, UK, 304016), were then added and incubated for 45 min at 4 °C. Cells were then washed twice in Perm Buffer I and then resuspended in wash buffer and acquired on FACS Canto II using FACS DIVA software. Data were then analysed using Flowjo software (Treestar, Ashland, OR, USA).

### 2.4. sCD163, Haptoglobin and Hemin Assays

Sputum supernatants processed with PBS were analysed for sCD163 (DuoSet ELISA, R and D Systems, Cambridge, UK) and haptoglobin (Quantikine ELISA, R and D Systems, Cambridge, UK) according to the manufacturer’s instructions.

### 2.5. Haptoglobin Phenotyping

Serum was diluted in PBS and heated for 15 min at 90 °C before being electrophoresed on 15% SDS/acrylamide gels, followed by a transfer onto a 0.2 µm nitrocellulose membrane (BioRad, Hertfordshire, UK) for 1 h at 4 °C. Membranes were blocked for 1 h in blocking buffer (5% milk in TBS, 0.1% Tween-20) before incubating with anti-haptoglobin (Sigma-Aldrich, Poole, UK HYB 170-06-02) diluted in block buffer overnight at 4 °C. Membranes were washed for 2 × 5 min in wash buffer (88mM Tris pH7.8, 0.1% Tween-20), prior to incubating with species-specific horse radish peroxidise conjugated goat anti-mouse secondary antibody (New England Biolabs, Hertfordshire, UK) for 1 h at room temperature. Membranes were washed (3 × 5 min) in wash buffer, and immunoreactive proteins were visualised using enhanced chemiluminesence in the BioRad Universal Hood II with Quantity One Software. Haptoglobin isolated from human plasma (Sigma-Aldrich, Poole, UK) was used as a reference, and protein molecular weights were determined using Precision Plus Standards (BioRad, Hertfordshire, UK).

### 2.6. CD163 Gene Expression

Lung macrophages were isolated from peripheral lung tissue of 21 patients, as previously described [25]. Following isolation, macrophages were left to adhere for 16 h prior to removal of non-adherent cells the following day. Culture supernatants were removed and cells were lysed in RLT buffer. Total RNA was purified from cell lysates using RNeasy kits (Qiagen, Crawley, UK) according to the manufacturer’s instructions. DNA contamination was prevented by an on-column addition of DNase (Qiagen, Crawley, UK) according to the manufacturer’s instructions. Reverse transcription was performed on 50 ng of RNA using the Verso cDNA kit (Thermo Fisher Scientific, Loughborough, UK). The resulting cDNA was reacted with ABsolute blue qPCR mix (Thermo Fisher Scientific, Loughborough, UK) in 25 µL reactions containing premade ABI Taqman gene expression assays for CD163 (Hs00174705_m1), and the endogenous control was glyceraldehyde-3phosphate dehydrogenase (GAPDH) (Catalogue no: 4352934E) (Applied Biosystems, Warrington, UK). Controls without RT-enzyme showed there was no genomic DNA amplification. Thermal cycling was carried out on a Stratagene MX3005P (Agilent Technologies, Cheadle, UK). Relative expression levels were determined using the 2^-ΔCt^.

### 2.7. Statistical Analysis

Statistical analyses were performed using GraphPad InStat software (GraphPad Software Inc, La Jolla, CA, USA). Data distributions were determined by the D’Agostino and Pearson normality test. Comparisons between three groups were made by a one-way ANOVA or Kruskal–Wallis with post-hoc tests. Comparisons between two groups were made by an unpaired *t*-test or Mann–Whitney test. Pearson correlations were performed to determine the associations between endpoints, presented as rho values. *p* values less than 0.05 were considered significant.

## 3. Results

### 3.1. Study Subjects

The clinical characteristics of the sputum study population are shown in Table 1. COPD patients were matched to controls for age and gender. Smoking pack-year history was higher in COPD patients compared to HS (*p* = 0.04). COPD patients had a significantly lower FEV1% predicted compared to HNS and HS (*p* < 0.0001). The percentage of sputum lymphocytes were significantly lower in COPD patients compared to HNS (*p* = 0.011), while there was a numerical trend for higher neutrophil percentages in COPD patients and HS (versus HNS) and higher eosinophil percentages in COPD. 

The clinical characteristics of the lung macrophage gene expression study population are shown in Table 2. There were no differences between COPDS and COPDE.

### 3.2. CD163 and sCD163 Measurements

**Figure 1:**
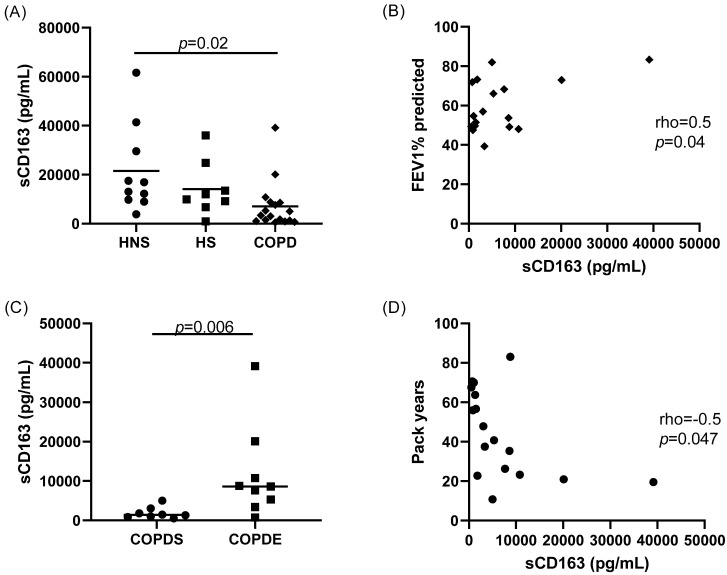
Soluble CD163 (sCD163) levels in sputum supernatants. The levels of sCD163 were quantified in the sputum supernatants of *n* = 10 healthy non-smokers (HNS), *n* = 10 healthy smokers (HS) and *n* = 17 COPD patients by ELISA (**A**). The relationship between sCD163 levels and forced expiratory volume in 1 s (FEV1)% predicted in COPD patients was examined (**B**). SCD163 levels were compared between *n* = 8 COPD current smokers (COPDS) and *n* = 9 COPD ex-smokers (COPDE) (**C**). The relationship between sCD163 levels and smoking pack-year history was examined in COPD patients (**D**). Data are presented as individual values with mean.

The levels of sCD163 were significantly lower in COPD patients compared to HNS (*p* = 0.02; Figure 1). There was a positive association between sCD163 and FEV1% predicted in COPD patients (rho = 0.5, *p* = 0.04; Figure 1) and when COPD patients and controls were combined (rho = 0.5, *p* = 0.0007; Figure S1). SCD163 levels were significantly lower in COPDS compared to COPDE (*p* = 0.006; Figure 1), and sCD163 levels were negatively associated with smoking pack-year history in COPD patients only (rho = −0.5, *p* = 0.047; Figure 1) and when HS were added (rho = −0.6, *p* = 0.004; Figure S1). 

**Figure 2:**
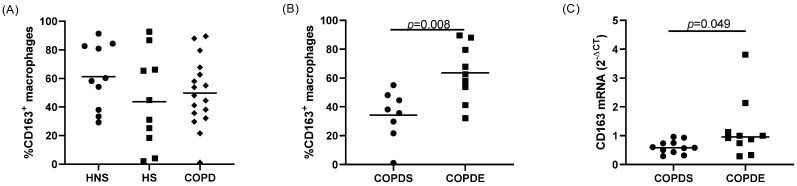
Macrophage CD163 protein and gene expression. The percentage of CD163+ macrophages were quantified in sputum from *n* = 10 healthy non-smokers (HNS), *n* = 10 healthy smokers (HS) and *n* = 17 COPD patients by flow cytometry (**A**). The percentage of CD163+ macrophages were compared between *n* = 8 COPD current smokers (COPDS) and *n* = 9 COPD ex-smokers (COPDE) (**B**). CD163 gene expression was quantified by real time PCR in macrophages isolated from the lung tissue of *n* = 11 COPDS and *n* = 10 COPDE (**C**). Data are presented as individual values with mean (**A**,**B**) or median (**C**).

The percentage of CD163+ sputum macrophages was not different between COPD patients and controls (Figure 2). However, the percentage of CD163+ sputum macrophages were significantly lower in COPDS compared to COPDE (*p* = 0.008; Figure 2). We also examined CD163 gene expression in lung macrophages isolated from the peripheral lung tissue of a separate group of COPD patients; CD163 gene expression was significantly lower in COPDS compared to COPDE (*p* = 0.049; Figure 2). 

There was no difference in sCD163 levels or CD163+ sputum macrophages when comparing COPD inhaled corticosteroid (ICS) users to non-users (Figure S2). 

Subgroup analyses according to current and ex-smoking status were limited by small sample sizes, and are shown in the online supplement.

### 3.3. Haptoglobin Measurements

**Figure 3:**
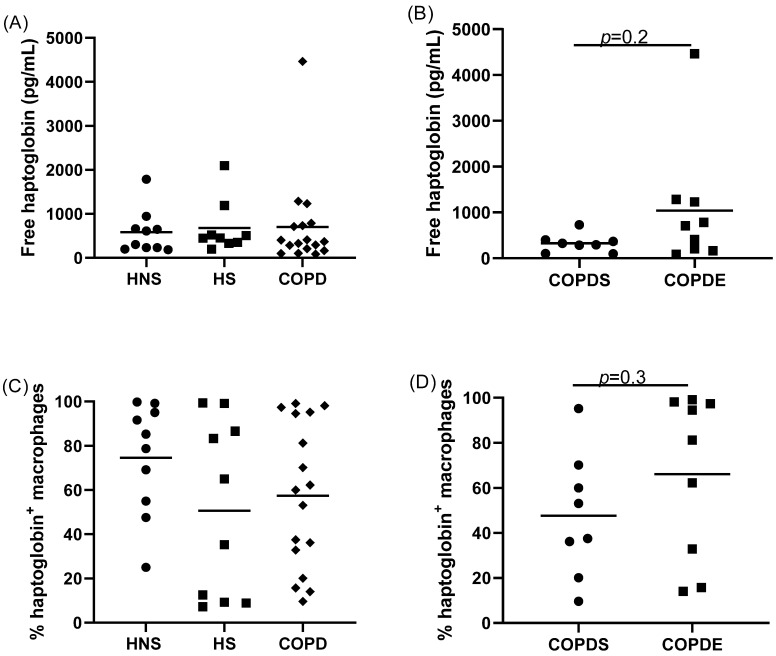
Sputum haptoglobin expression. The levels of haptoglobin were quantified in the sputum supernatants of *n* = 10 healthy non-smokers (HNS), *n* = 10 healthy smokers (HS) and *n* = 17 COPD patients by ELISA (**A**). Haptoglobin levels were compared between *n* = 8 COPD current smokers (COPDS) and *n* = 9 COPD ex-smokers (COPDE) (**B**). The percentage of haptoglobin+ sputum macrophages were compared between HNS, HS and COPD patients (**C**) and between COPDS and COPDE (**D**). Data are presented as individual values with mean.

The levels of free haptoglobin in the sputum supernatants of COPD patients were similar to controls (Figure 3). There was a numerical trend for reduced free haptoglobin in COPDS compared to COPDE, which was not significant (*p* = 0.2; Figure 3). 

There was a numerical reduction in the percentage of haptoglobin+ sputum macrophages in COPD patients (57%) and HS (51%) compared to HNS (75%), but this was not significant (ANOVA, *p* = 0.25; Figure 3). Similarly, there was a numerical reduction in the percentage of haptoglobin+ sputum macrophages in COPDS compared to COPDE which was not significant (*p* = 0.3; Figure 3).

ICS use in COPD patients had no effect on free haptoglobin levels or haptoglobin+ sputum macrophages (Figure S3). 

It has been reported that lung tissue eosinophils express haptoglobin mRNA, and so we examined haptoglobin protein expression in sputum eosinophils [15]. There were no differences in the percentage of haptoglobin+ eosinophils between COPD and controls (Figure S3). Likewise, there were no differences between COPDS vs. COPDE and ICS users vs. non-users (Figure S4).

**Figure 4:**
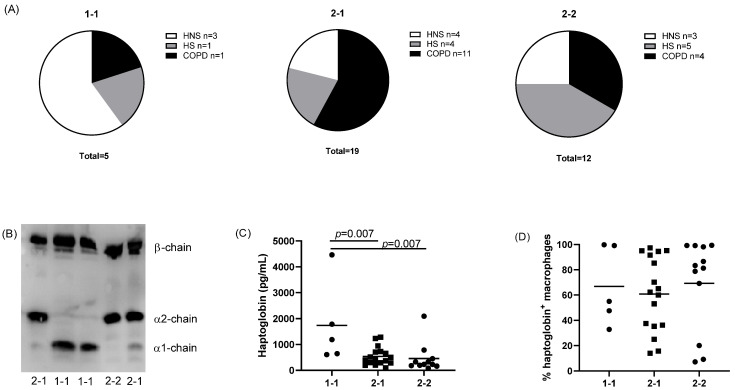
Haptoglobin genotype in the study population. The numbers of individuals with genotype 2-1 or 2-2 are presented (**A**) with a representative blot indicating genotype identification in serum samples (**B**). The levels of haptoglobin in sputum supernatants (**C**) and haptoglobin+ sputum macrophages (**D**) were compared between the different genotype groups. Data are presented as individual values with mean.

Haptoglobin levels are influenced by genotype [9,10]. We separated patients into genotype groups (Figure 4). The levels of haptoglobin in the sputum supernatants of individuals with the genotype 1-1 (*n* = 5; 14% of total study population) were significantly higher than 2-1 (*n* = 19; 53%) or 2-2 (*n* = 12; 33%) individuals (*p* = 0.007 for both comparisons; Figure 4). There was no difference in the percentage of haptoglobin+ macrophages between individuals with different genotypes (Figure 4).

### 3.4. Relationship between CD163 and Haptoglobin

**Figure 5:**
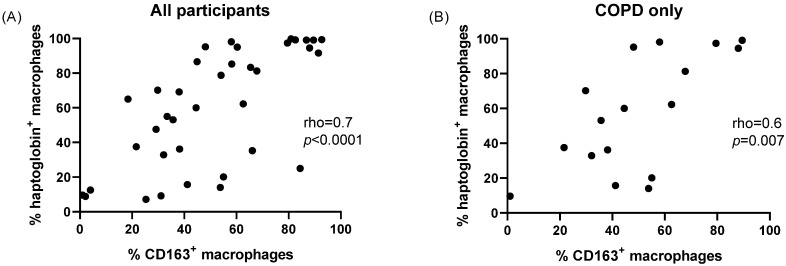
Relationship between haptoglobin and CD163 expression in the study population. The numbers of haptoglobin+ and CD163+ sputum macrophages were quantified by flow cytometry and the relationship examined by a Pearson’s correlation in the total study population (**A**) and in COPD patients only (**B**).

There was a positive correlation between the percentage of haptoglobin+ macrophages and the percentage of CD163+ macrophages when analysing all participants (rho = 0.7, *p* < 0.0001) and COPD patients only (rho = 0.6, *p* = 0.007) (both Figure 5).

## 4. Discussion

We have shown that current smoking and the presence of COPD downregulate sCD163 levels in sputum supernatants. As sputum macrophage CD163 protein expression levels were not significantly different between COPD patients and controls, then decreased sCD163 levels cannot be explained by altered CD163 protein levels. Potential explanations include reduced CD163 cleavage or decreased release of sCD163 in extracellular vesicles in COPD patients. Haptoglobin protein levels were not modified in COPD patients compared to controls but were very clearly related to previously described genotypes [9,10].

The rationale for this study was to evaluate dysregulation of the CD163-haptoglobin axis in COPD, as these proteins regulate iron bioavailability following red cell lysis [12]. The number of iron positive alveolar macrophages is increased in COPD patients compared to controls, with a positive association to disease severity [27]. This suggests that increased iron bioavailability is linked to worse disease outcomes. We now show that sputum sCD163 levels are reduced in COPD patients and are negatively correlated with FEV1% predicted. These findings implicate sCD163 levels in disease pathophysiology, presumably because lower levels will lead to greater extracellular iron levels [23].

Haemoglobin is a potent source of labile iron (Fe2+), which is redox active [28]. Via the Fenton reaction, labile iron catalyses the production of reactive oxygen species, including lipid peroxides [29]. Lipid peroxides are highly cytotoxic, disrupting the cell membrane and causing DNA and protein damage [28]. This can lead to ferroptosis, which is an iron-dependent regulated cell death due to increased lipid peroxidation [29]. Recently, it has been shown that free iron is increased in COPD bronchial epithelial cells, along with increased markers of ferroptosis (shrunken mitochondria and increased membrane density) and increased lipid peroxides in lung homogenate from COPD patients [30]. Using cell culture and animal models, the authors showed that cigarette smoke increased labile iron and induced ferroptosis. Our findings implicate reduced sCD163 levels as a mechanism that can promote higher extracellular iron levels in COPD patients and the subsequent sequelae including ferroptosis.

There is also evidence of increased iron accumulation in the lung tissue of idiopathic pulmonary fibrosis patients, and this was associated with worse lung function [31]. Furthermore, the authors demonstrated increased fibroblast proliferation, increased collagen 1 and tenascin C mRNA levels, and increased IL-6 and CXCL8 release following ferric ammonium citrate (source of iron) exposure. Small airway fibrosis is a component of COPD pathophysiology and perhaps increased extracellular iron accumulation due to reduced sCD163 expression may play a role in this regard.

Whilst we observed reduced macrophage CD163 protein expression in COPDS compared to COPDE, CD163 expression was not different in COPD patients compared to controls. Therefore, we now speculate on alternative mechanisms to explain reduced sCD163 in COPD patients. ADAM17 regulates CD163 cleavage from the surface membrane. The expression of ADAM17 has not been determined in COPD macrophages compared to controls, but there is evidence for increased numbers of cells (presumably immune cells) positive for ADAM17 phosphorylation at 735-threonine (and hence increased activation) in the alveolar spaces of COPD patients with emphysema compared to controls [32]. However, animal model data are conflicting with one study suggesting a protective role of ADAM17 in emphysema development [33], whereas another study suggests ADAM17 activity promotes emphysema development [32]. Future studies should examine ADAM17 expression and activity in COPD macrophages. Extracellular vesicle associated CD163 also contributes to overall sCD163 levels [22], raising the possibility of dysregulation of the release of extracellular vesicle-associated CD163 contributing to reduced sCD163 in COPD.

We observed reduced sCD163 levels and macrophage CD163 protein and gene expression in COPDS compared to COPDE, along with a negative correlation to pack-year history. This aligns to previous data and indicates a role for cigarette smoking in regulating CD163 expression [18,19]. The cellular signalling pathways involved in this are unclear, but our data suggests that inhibition of gene expression contributes to reduced CD163 expression in COPD current smokers.

Haptoglobin gene expression has been observed in pulmonary and blood eosinophils [15]. We now extend these observations and confirm pulmonary eosinophils express haptoglobin at the cell surface. The role for haptoglobin in eosinophil function is unclear. However, we have previously demonstrated increased haptoglobin levels in the BAL fluid of eosinophil^high^ compared to eosinophil^low^ COPD patients [34]. Eosinophil-derived haptoglobin may be contributing to these observations. Interestingly, COPD patients with higher numbers of eosinophils have lower levels of pathogenic bacteria in the airways [35,36]. Iron is an essential nutrient for bacterial growth. Perhaps increased haptoglobin levels increase CD163-haptoglobin mediated uptake of haemoglobin and thus restrict iron for bacterial growth in these patients.

It has previously been shown that serum haptoglobin levels are higher in individuals with the genotype 1-1 compared to 2-1 and 2-2 individuals [9,10]. For the first time, we show that pulmonary haptoglobin levels follow the same pattern. The percentage of individuals in our overall study population with genotypes 1-1, 2-1 and 2-2 was 14%, 53% and 33%. This is in agreement with previous data from a larger UK study (*n* = 218) [26]. Additionally, there was a correlation between cellular haptoglobin levels and macrophage CD163 expression, in keeping with the known role of CD163 with regard to facilitating cellular haptoglobin uptake [22,23]. There was no alteration of haptoglobin levels in COPD compared to controls, and overall, we conclude that pulmonary levels of free haptoglobin are predominantly under genotype control, while cellular haptoglobin levels are associated to CD163 levels rather than genotype.

There are some limitations to our study. Firstly, sample numbers were relatively limited, which constrains the statistical power of the study. Nevertheless, some of our data are very consistent with previous findings (e.g., smoking effect on macrophage CD163 expression, genotype control of haptoglobin expression, proportion of haptoglobin genotypes in the study population), indicating robustness of data despite sample size. Secondly, our results are cross-sectional. It would be interesting to examine sCD163 expression longitudinally and monitor the relationship with lung function decline in COPD patients. It would also be interesting to examine the relationship between the CD163-haptoglobin axis and bacterial colonisation in COPD, particularly during exacerbations. Whilst we have studied the levels of haptoglobin in sputum, it would also be important to measure the levels of haemoglobin and free iron for completeness, which could be a focus of future studies.

## 5. Conclusions

In conclusion, we have shown that COPD and current smoking reduce sCD163 levels in sputum supernatants. These data indicate a dysfunctional CD163-haptoglobin axis in COPD, which may contribute to disease pathophysiology, presumably due to the harmful effects of reduced clearance of extracellular iron.

## Figures and Tables

**Table 1:** Clinical characteristics of the sputum study population.

	Non-Smoker	Smoker	COPD	ANOVA *p* Value
*n*	10	10	17	n/a
Age (Years)	62 (7)	61 (9)	68 (7)	0.08
Gender: F/M	5/5	5/5	7/10	0.9
Current smokers (*n*)	0	3	8	0.4
Pack years	n/a	27 (12) *	43 (21)	0.04
BMI (kg/m^2^)	29 (4)	28 (4)	29 (5)	0.6
Exacerbation rate (1 years period)	n/a	n/a	0.9 (0.8)	n/a
FEV1 (L)	3.1 (0.8) ***	3.0 (0.6) ***	1.6 (0.5)	<0.0001
FEV1% predicted	112 (11) ***	109 (17) ***	60 (13)	<0.0001
FEV1/FVC ratio	0.8 (0.04) ***	0.8 (0.04) ***	0.5 (0.1)	< 0.0001
GOLD stage	n/a	n/a		n/a
1			2	
2			10	
3			5	
4			0	
CAT	n/a	n/a	14 (10)	n/a
mMRC	n/a	n/a	1.8 (1.2)	n/a
SGRQ (total)	n/a	n/a	36 (24)	n/a
Atopy positive (*n*)	2	1	1	0.5
Chronic bronchitis (*n*)	n/a	n/a	13	n/a
ICS users (*n*)	n/a	n/a	10	n/a
LAMA users (*n*)	n/a	n/a	15	n/a
LABA users (*n*)	n/a	n/a	11	n/a
No inhaled medication (*n*)	n/a	n/a	1	n/a
Sputum Characteristics				
Macrophage (%)	32 [18–66]	18 [7–47]	16 [2–67]	0.2
Neutrophil (%)	63 [27–78]	79 [46–91]	70 [28–96]	0.2
Eosinophil (%)	0.3 [0–0.8]	0.4 [0–2.0]	0.8 [0–15.3]	0.08
Lymphocyte (%)	0.8 [0–2.5] *	0.6 [0–1.0]	0.3 [0–0.5]	0.049
Epithelial (%)	4 [0–14]	2 [1–7]	4 [0–15]	0.3

BMI, body mass index; CAT, COPD assessment test; FEV1, forced expiratory volume in 1 s; FVC, forced vital capacity; ICS, inhaled corticosteroids; LABA, long-acting beta agonist; LAMA, long-acting muscarinic antagonist; mMRC, modified medical research council questionnaire; SGRQ, St George’s respiratory questionnaire. Date presented as mean (standard deviation) or median [range]. *p* < 0.05 was considered significant; * = significant difference vs. COPD patients following a post-hoc test when ANOVA *p* value was less than 0.05. One symbol *p* < 0.05, and three symbols *p* < 0.001.

**Table 2:** Clinical characteristics of the lung macrophage study population.

	COPDS	COPDE	*p* Value
*n*	10	17	n/a
Age (Years)	69 (5)	70 (5)	0.5
Gender: F/M	7/4	7/3	0.8
Pack years	68 (53)	38 (12)	0.1
FEV1 (L)	1.9 (0.5)	1.8 (0.4)	0.6
FEV1% predicted	85 (15)	85 (23)	1.0
FEV1/FVC ratio	65 (7)	61 (10)	0.4
GOLD stage			
1	6	6	0.8
2	5	4	0.7
3	0	1	0.7
4	0	0	n/a
ICS users (*n*)	4	2	0.4
LAMA users (*n*)	2	4	0.3
LABA users (*n*)	0	0	n/a
No inhaled medication (*n*)	6	5	0.8

FEV1, forced expiratory volume in 1 s; FVC, forced vital capacity; ICS, inhaled corticosteroids; LABA, long-acting beta agonist; LAMA, long-acting muscarinic antagonist.

## Data Availability

The datasets generated and/or analysed during the current study are not publicly available.

## Supplementary Materials

The following are available online at https://www.mdpi.com/article/10.3390/cells11010002/s1, Figure S1: The relationship between forced expiratory volume in 1 s (FEV1)% predicted and pack-year history with sputum sCD163 levels in COPD patients and controls. Figure S2: Sputum sCD163 levels and macrophage CD163 expression in ICS users and non-users. Figure S3: Sputum haptoglobin levels and macrophage haptoglobin expression in ICS users and non-users. Figure S4: Haptoglobin expression in sputum eosinophils.
