# Supplementary material for: Dysregulation of the CD163-Haptoglobin Axis in the Airways of COPD Patients
Source: Cells. 2021 Dec 21;11(1):2. doi: 10.3390/cells11010002 (PMC8750523; doi:10.3390/cells11010002)

Supplementary Material

Dysregulation of the CD163-haptoglobin axis in the airways of COPD patients

Andrew Higham[1], James Baker[1], Natalie Jackson[2], Rajesh Shah[3], Simon Lea[1] and Dave Singh[1,2]

1. The University of Manchester Division of Infection, Immunity and Respiratory Medicine, School of Biological Sciences, Faculty of Biology, Medicine and Health, Manchester Academic Health Science Centre, The University of Manchester and University Hospital of South Manchester, NHS Foundation Trust, Manchester, UK.
2. Medicines Evaluation Unit, The Langley Building, Southmoor Road, The University Hospital of South Manchester, Manchester, UK.
3. Manchester University Hospital NHS Foundation Trust, Department of Thoracic Surgery

Supplementary results

There was a numerical trend for reduced sCD163 levels in COPDS compared to healthy current smokers (S) and COPDE compared to healthy ex-smokers (E) but this did not reach statistical significance (p=0.3 and p=0.2 respectively; Figures S5 and S6). There was no difference in sCD163 levels when comparing S to E (p=0.9; Figure S7).

There were no significant differences in the percentage of CD163+ macrophages when comparing COPDS to S, COPDE to E or S to E. (p=0.3 – 0.9; Figures S5–S7).

There were no significant differences in the levels of free haptoglobin when comparing COPDS to S, COPDE to E or S to E. (p=0.2 – 0.9; Figure S5–S7).

Supplementary discussion

We observed a non-significant reduction in sCD163 levels when comparing COPDS to S and COPDE to E. These sub analyses were limited by small sample sizes. Although not significant, this observation supports the concept of a dysfunctional CD163-haptolgobin axis in COPD. In addition, we didn't observe a difference between S and E, although these subgroup analyses were limited by low sample numbers.

Supplementary figure legends

Supplementary Figure S1. The relationship between sCD163 and clinical variables. The relationship between sCD163 in sputum supernatants and forced expiratory volume in 1 second (FEV1) % predicted in the total population (n=10 healthy non-smokers (HNS), n=10 healthy smokers (HS) and n=17 COPD patients) was examined by Pearson's correlation (A). The relationship between sCD163 levels and smoking pack years history in HS and COPD patients was examined by Pearson's correlation (B).

Supplementary Figure S2. CD163 expression in inhaled corticosteroid users vs non-users. SCD163 levels in sputum supernatants (A) and the percentage of $CD163^+$ sputum macrophages (B) were compared between COPD patients who use inhaled corticosteroids (ICS yes) and those who do not (ICS no). Data presented as individual values with mean.

Supplementary Figure S3. Haptglobin expression in inhaled corticosteroid users vs non-users. Haptoglobin levels in sputum supernatants (A) and the percentage of $haptoglobin^+$ sputum macrophages (B) were compared between COPD patients who use inhaled corticosteroids (ICS yes) and those who do not (ICS no). Data presented as individual values with mean.

Supplementary Figure S4. Haptoglobin expression in sputum eosinophils. The percentage of $haptoglobin^+$ eosinophils was compared between COPD patients and controls (A), COPD current smokers (COPDS) and COPD ex-smokers (COPDE) (B) and COPD patients who use inhaled

corticosteroids (ICS yes) and those who do not (ICS no) (C). Data presented as individual values with mean.

Supplementary Figure S5. Comparison between healthy current smokers (S) and COPD current smokers (COPDS). SCD163 levels (A) the percentage of $CD163^{+}$ sputum macrophages (B) and free haptglobin levels (C) were compared between S (n=3) and COPDS (n=8). Data presented as individual values with median.

Supplementary Figure S6. Comparison between healthy ex-smokers (E) and COPD ex- smokers (COPDE). SCD163 levels (A) the percentage of $CD163^{+}$ sputum macrophages (B) and free haptglobin levels (C) were compared between E (n=7) and COPDS (n=9). Data presented as individual values with median.

Supplementary Figure S7. Comparison between healthy current smokers (S) and healthy ex- smokers (E). SCD163 levels (A) the percentage of $CD163^{+}$ sputum macrophages (B) and free haptglobin levels (C) were compared between S (n=3) and E (n=7). Data presented as individual values with median.

Supplementary Figure S1

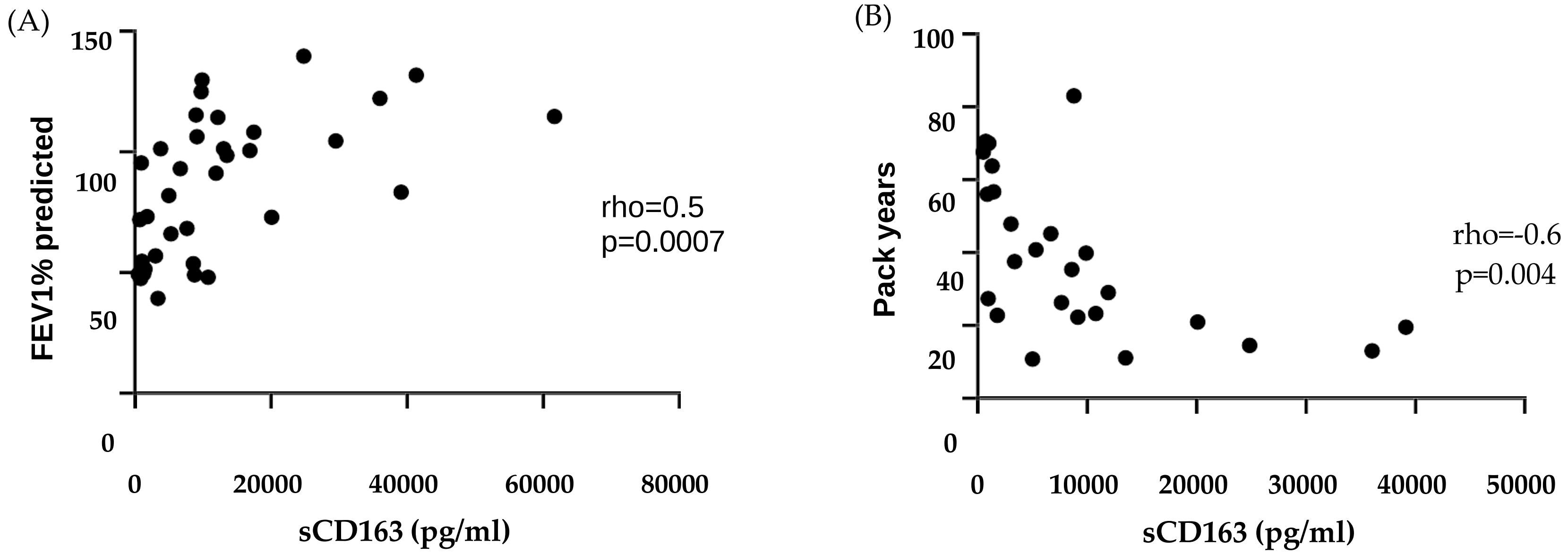

Supplementary Figure S2

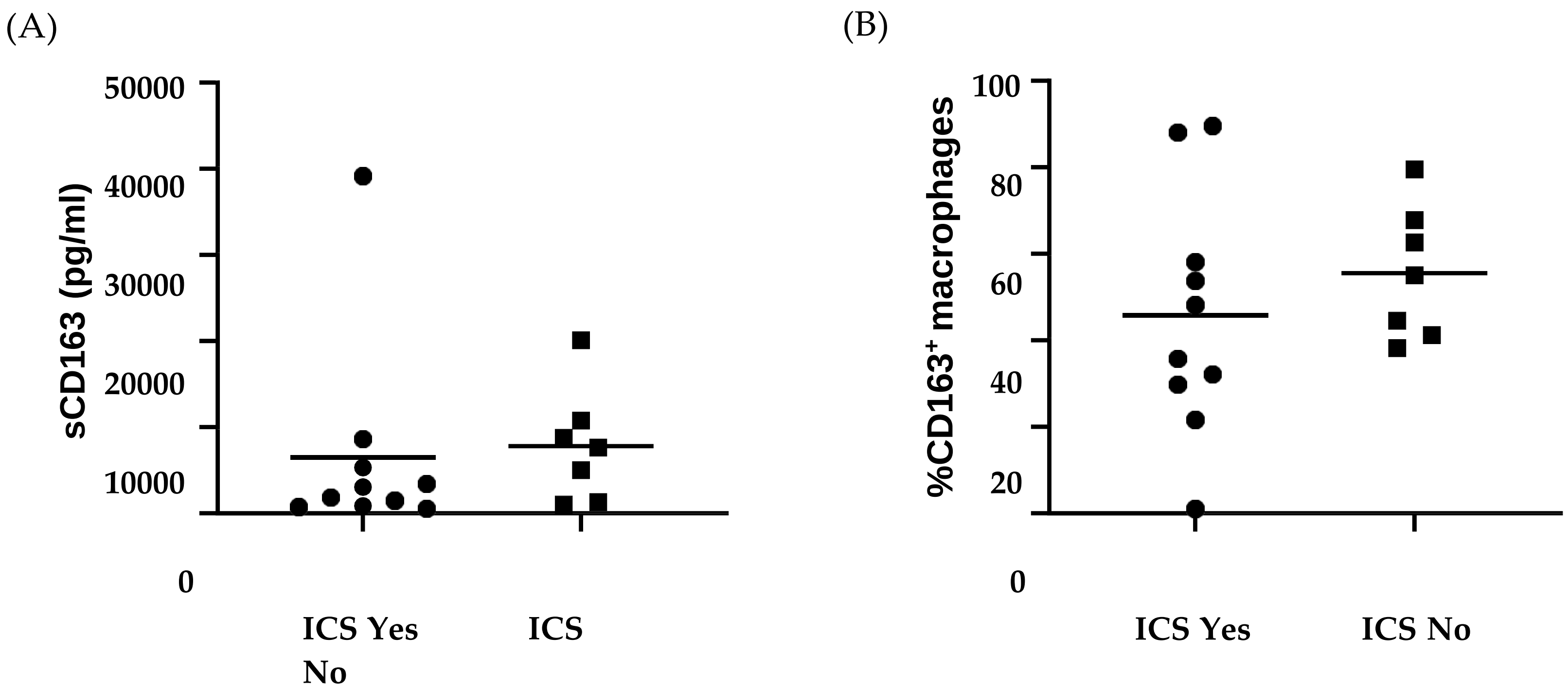

Supplementary Figure S3

(A)

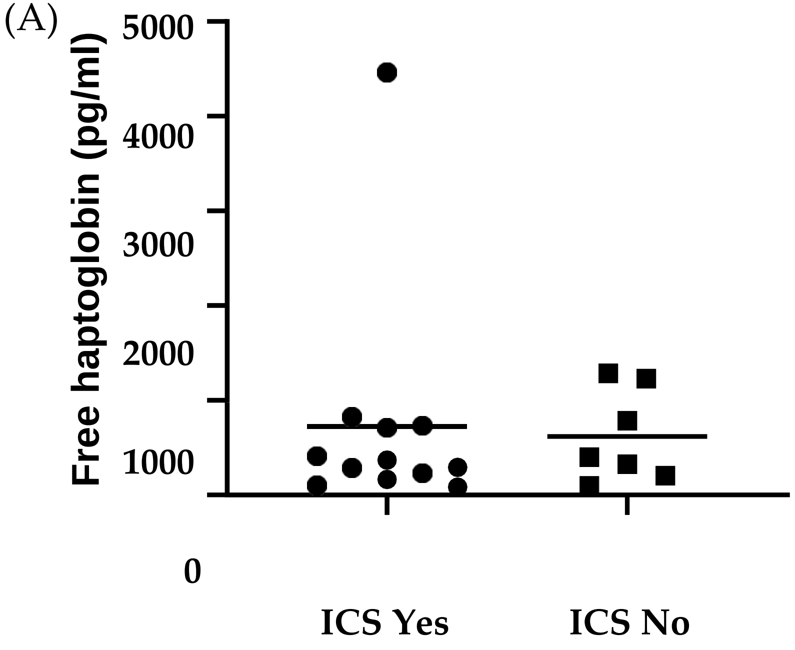

(B)

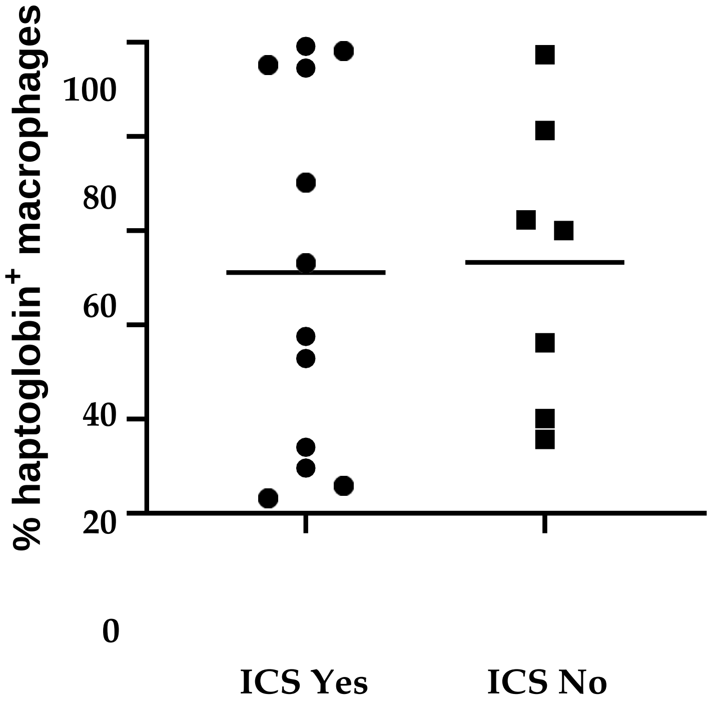

Supplementary Figure S4

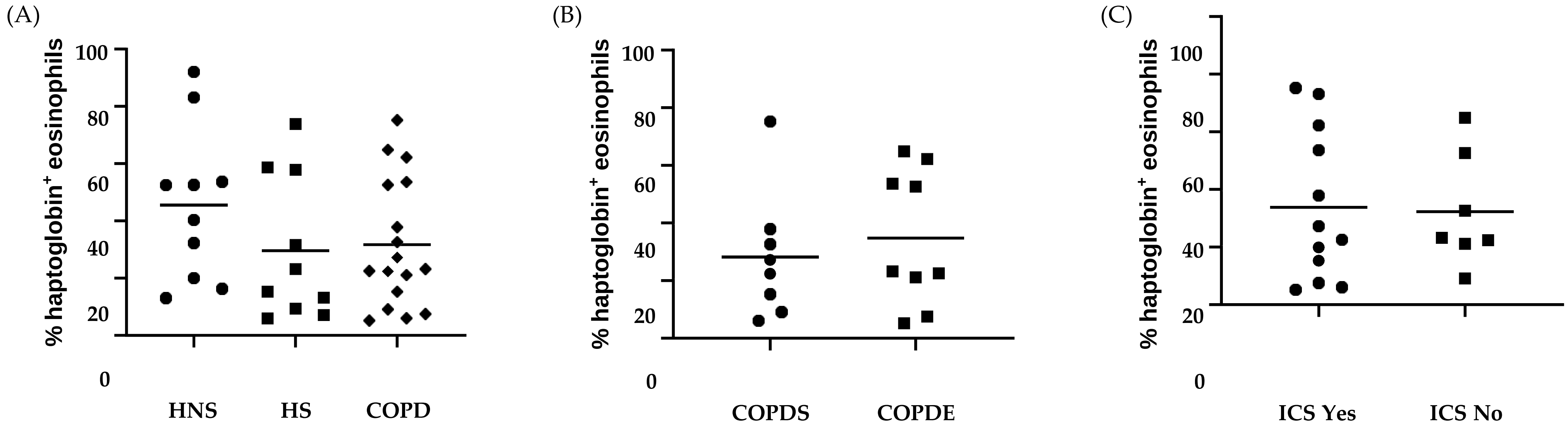

Supplementary Figure S5

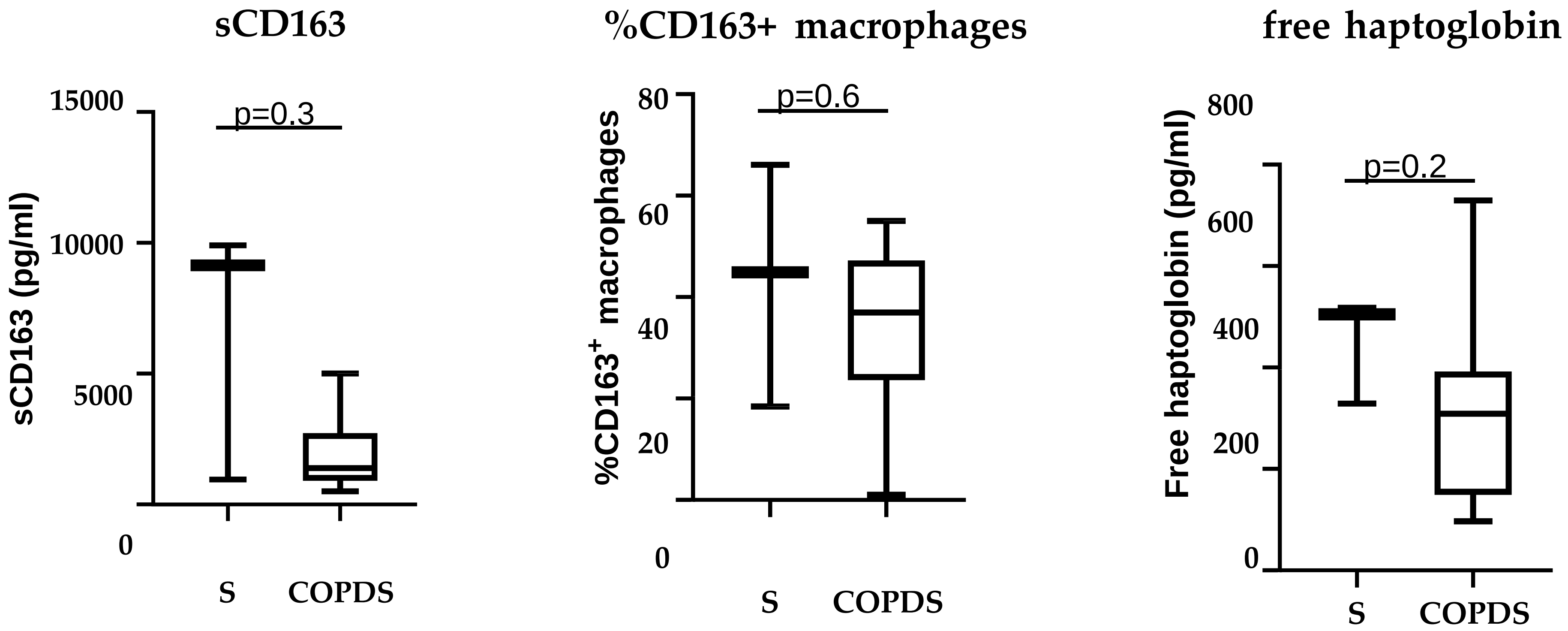

Supplementary Figure S6

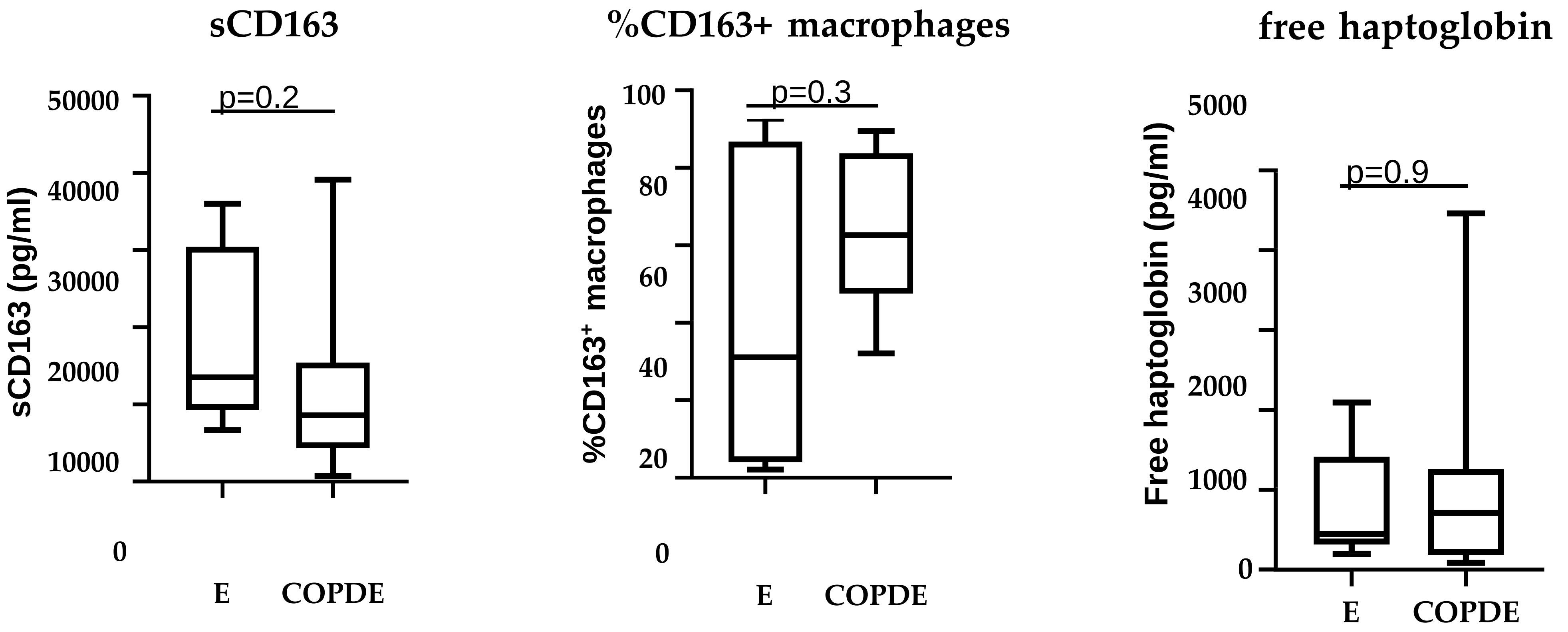

Supplementary Figure S7

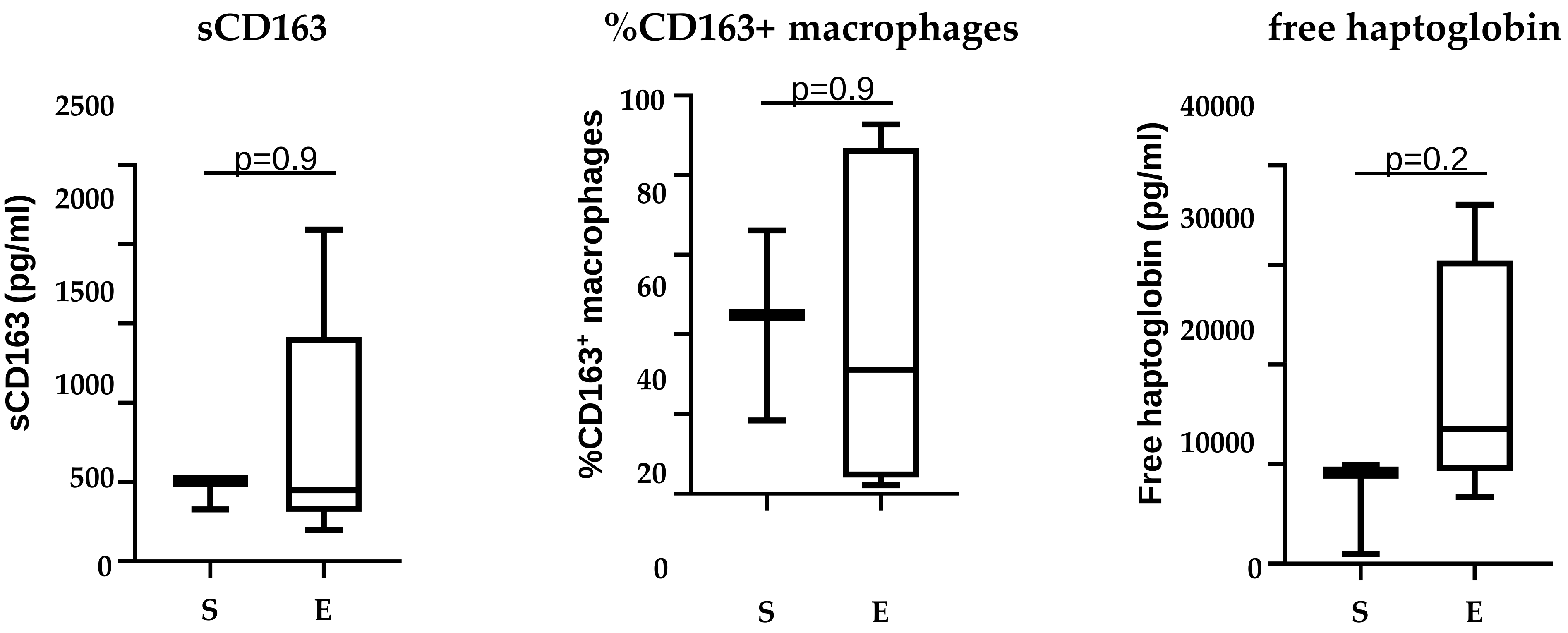

Supplement: Supplementary file 1 [file cells-11-00002-s001.zip › cells-1491203-supplementary.pdf]
